# Validation of a novel real-time PCR for detecting *Rasamsonia argillacea* species complex in respiratory secretions from cystic fibrosis patients

**DOI:** 10.1002/nmi2.44

**Published:** 2014-04-20

**Authors:** J Steinmann, S Giraud, D Schmidt, L Sedlacek, A Hamprecht, J Houbraken, J F Meis, J P Bouchara, J Buer, P-M Rath

**Affiliations:** 1 Institute of Medical Microbiology, University Hospital Essen, University of Duisburg-EssenEssen, Germany; 2 L'UNAM Université, Université d′Angers, Groupe d′Etude des Interactions Hôte-PathogèneAngers, France; 3 Institute of Medical Microbiology and Hospital Epidemiology, Hannover Medical SchoolHannover, Germany; 4 Institute for Medical Microbiology, Immunology and Hygiene, University Hospital of CologneCologne, Germany; 5 CBS-KNAW Fungal Biodiversity CentreUtrecht, The Netherlands; 6 Department of Medical Microbiology and Infectious Diseases, Canisius Wilhelmina HospitalNijmegen, The Netherlands; 7 Department of Medical Microbiology, Radboud University Medical CentreNijmegen, The Netherlands; 8 Laboratory of Parasitology and Mycology, Angers University HospitalAngers, France

**Keywords:** Cystic fibrosis, *Geosmithia*, PCR, *Rasamsonia*

## Abstract

Members of the recently introduced fungal genus *Rasamsonia* (formerly included in the *Geosmithia* genus) have been described as emerging pathogens in immunosuppressed hosts or patients with cystic fibrosis (CF). *Rasamsonia* species have often been misidentified as *Penicillium* or *Paecilomyces* because of similar morphological characteristics. We validated a commercially available real-time PCR assay (Primerdesign™, UK) for accurate detection of species from the *Rasamsonia argillacea* complex. First, we tested this assay with a collection of 74 reference strains and clinical isolates and then compared the PCR with cultures of 234 respiratory samples from 152 patients with CF from two University Hospitals in Germany and France. The assay reliably detected the three main species within the *Rasamsonia argillacea* species complex (*R. argillacea*, *R. piperina*, *R. aegroticola*), which are typically encountered in CF patients. The limit of DNA detection was between 0.01 and 1 pg/μL. Analysis of the DNA extracts from respiratory specimens of CF patients revealed that four out of the 153 patients studied (2.6%) were colonized with *R. argillacea* species complex. Two species from the *R. argillacea* complex grew in the parallel cultures from the same patients. In one patient the PCR was positive 5 months before culture. The real-time PCR assay is a sensitive and specific method for detecting the three most important species of the *R*. *argillacea* species complex encountered in the CF context. Detection of these emerging pathogens in respiratory secretions from CF patients by this novel assay may increase our understanding of the occurrence and epidemiology of the *R. argillacea* species complex.

## Introduction

Cystic fibrosis (CF) is an autosomal recessive disease caused by mutations in the cystic fibrosis transmembrane regulator (*CFTR*) gene. This disease occurs in ∼ 1 in 2500 live births in the Caucasian population 1 and affects exocrine glands of several organs, particularly the lungs, where it results in the production of thick and sticky mucus. In the lower airways, this provides an ideal breeding ground for many microorganisms and facilitates recurrent respiratory infections with viruses, bacteria and fungi 2. *Aspergillus fumigatus* is the most prevalent filamentous fungus in the respiratory tract of CF patients 3. It has been reported that CF patients chronically colonized with *A. fumigatus* have a decreased lung function, more frequent exacerbations and more prominent radiological abnormalities than non-colonized or transiently colonized CF patients 4. Besides *Aspergillus* spp., *Scedosporium/Pseudallescheria* species were reported ranking usually second among the filamentous fungi colonizing the CF airways 5–7. Also other moulds like *Aspergillus terreus* or *Exophiala dermatitidis* may also be encountered in the CF context, with prevalence rates varying from one country to another. However, the clinical relevance of the airway colonization by non-*A. fumigatus* moulds is not clearly established. Recently, a new species complex, *R. argillacea*, has been described as emerging in patients with CF 8,9.

*Rasamsonia* spp. are filamentous thermophilic fungi commonly isolated from hot environments 10,11. The species *R. argillacea* was first described by Stolk *et al*. in 1969 under the name *Penicillium argillaceum* 12. Ten years later, Pitt renamed this species *Geosmithia* because of its morphological characteristics 13. In 1999, this fungus was described for the first time in a French CF patient as *P. emersonii* based on morphological characteristics 14. In 2009, researchers reported the first isolation of this organism from a disseminated infection in a German Shepherd dog 15. Recently, Houbraken *et al*. showed that *G. argillacea* and other *Geosmithia* species form a distinct clade within the Trichocomaceae 16 and proposed the new genus *Rasamsonia*. In addition, phylogenetic analyses of the internal transcribed spacer, partial *β*-tubulin and calmodulin sequences revealed that *R. argillacea* is a species complex comprising four distinct species 16. Re-identification of nine published CF isolates from France revealed three cases of *R. argillacea*, four colonizations with *R. aegroticola*, and two cases of *R. piperina* 8,16. In contrast, *R*. *eburnea* has also been found in sterile fluids of human origin but not in patients with CF 16.

In the present study, we validated a novel real-time PCR assay (PrimerDesign™, Southampton, UK) for the detection of the three species of the *R. argillacea* species complex typically found in CF patients and compared the results of this assay with cultures of reference strains. In addition, we evaluated the performance of this novel PCR assay by testing respiratory samples from patients with CF.

## Methods

### Culture isolates

In order to assess the specificity of the real-time PCR assay, 39 reference strains and clinical isolates from non-*Rasamsonia* species from the fungal and bacterial culture collection of the Institute of Medical Microbiology (IMMI), University Hospital Essen, Essen, Germany were analysed (Table1).

**Table 1:** Non-*Rasamsonia* strains (*n* = 39) tested for cross-reactivity in the *Rasamsonia argillacea* species complex PCR

Species	Strain number
Fungi	
*Aspergillus* cf. *tamarii*	ATCC 64841
*Aspergillus flavus*	CBS 113.49
*Aspergillus fumigatus*	NCPF2140
*A. fumigatus*	CBS 133.61
*A. fumigatus*	CBS 154.89
*Aspergillus nidulans*	CBS 100.20
*Aspergillus niger*	CBS 112.30
*Aspergillus brasiliensis*	CBS 733.88
*Aspergillus terreus*	CBS 469.81
*Aspergillus versicolor*	IMMI F81
*Candida albicans*	ATCC 44374
*Candida glabrata*	DSM 70614
*Candida guilliermondii*	ATCC 90877
*Candida kefyr*	DSM1195
*Candida krusei*	DSM 70075
*Candida parapsilosis*	ATCC 22019
*Candida tropicalis*	ATCC 750
*Exophiala dermatitidis*	CBS 120550
*Fusarium solani*	IMMI 1650
*Paecilomyces lilacinus*	IMMI 1900
*Penicillium citrinum*	IMMI 1945
*Penicillium notatum*	IMMI 2013
*Pseudallescheria boydii*	FMR 84
*Rhizomucor microsporus*	IMMI 1672
*Rhizomucor pusillus*	IMMI 1671
*Scedosporium apiospermum*	IMMI F71
*Scedosporium prolificans*	IMMI F78
Bacteria	
*Enterococcus faecalis*	ATCC 29212
*Enterococcus faecium*	DSM 13590
*Staphylococcus aureus*	ATCC 43300
*Staphylococcus epidermidis*	DSM 1789
*Streptococcus pyogenes*	DSM 11728
*Acinetobacter baumannii*	IMMI 150
*Enterobacter cloacae*	IMMI 253
*Haemophilus influenzae*	DSM 9999
*Klebsiella pneumoniae*	IMMI 251
*Legionella pneumophila*	ATCC 33152
*Proteus mirabilis*	DSM 4479
*Serratia marcescens*	ATCC 13880
*Mycobacterium tuberculosis*	ATCC 27294
*Mycobacterium avium*	DSM 44156

NCPF, national collection of pathogenic fungi; CBS, culture collection of the CBS-Fungal Biodiversity Centre, Utrecht, the Netherlands; ATCC, American Type Culture Collection; IMMI, internal fungal collection of the Institute of Medical Microbiology, University Hospital Essen, Essen, Germany; FMR, Facultat de Medicina, Reus, Spain.

Twenty-two reference strains belonging to the genus *Rasamsonia* were obtained from the collection of the Centraalbureau voor Schimmelcultures (CBS)-Fungal Biodiversity Centre, Utrecht, the Netherlands (DTO) (Table2). Thirteen clinical isolates were collected from clinical specimens submitted to three German university hospitals in 2010 and 2011 (Hannover, Cologne, Essen) (Table2).

**Table 2:** *Rasamsonia* culture isolates (*n* = 35) used for validation of the *Rasamsonia argillacea* species complex PCR

Species	Strain no.	Source of isolation	Origin
*Rasamsonia* reference strains (*n* = 22)			
*R. aegroticola*	DTO 049D4	Sputum from CF patient	UK
*R. aegroticola*	DTO 137A8 = CBS 132819	Respiratory secretion from CF patient	France
*R. argillacea*	CBS 128787	Heat-treated fruit concentrate	The Netherlands
*R. argillacea*	DTO 097E4	Mine tip	UK
*R. argillacea*	DTO 097E5	Air	UK
*R. argillacea*	DTO 097E7	Unknown source	UK
*R. brevistipitata*	DTO 25H2	Indoor environment of school	Germany
*R. brevistipitata*	DTO 26B1	Indoor environment of school	Germany
*R. cylindrospora*	DTO 138F7	Sputum	The Netherlands
*R. cylindrospora*	DTO 138F8	Culture contaminant	UK
*R. eburnea*	DTO 045I3	Contaminant of blood culture	UK
*R. eburnea*	DTO 049D9	Peritoneal dialysis fluid	UK
*R. piperina*	DTO 076F1	Seed of *Piper nigrum*	Spain
*R. piperina*	DTO 097E6	Wood chips of *Picea abis*	Sweden
*R. piperina*	DTO 097E9	Bronchial washing	Canada
*R. piperina*	DTO 138F5	Necropsy thoracic vertebra of dog	USA
*R. piperina*	DTO 138F6	Necropsy thoracic vertebra of dog	USA
*R. piperina*	DTO 139F9	Air	Germany
*R. piperina*	DTO 138G1	Wood chips of *Picea abies*	Sweden
*R. piperina*	DTO 138G2	Wood chips of *Picea abies*	Sweden
*R. piperina*	DTO 138G3	Seed of *Piper nigrum*	Spain
*R. piperina*	CBS 128034	Necropsy ex vertebra of canine	USA
*Rasamsonia* clinical isolates (*n* = 13)			
*R. aegritocola*	IMMI 1419	BAL from lung transplant recipient	Germany
*R. aegritocola*	IMMI 1603	Bronchial secretion from lung transplant recipient	Germany
*R. aegroticola*	IMMI 1869	BAL from lung transplant recipient	Germany
*R. aegroticola*	IMMI 1896	Sputum from CF patient	Germany
*R. aegroticola*	IMMI 2824	Sputum from CF patient	Germany
*R. argillacea*	IMMI 1764	BAL from bone marrow transplant recipient	Germany
*R. argillacea*	IMMI 1862	Sputum from CF patient	Germany
*R. argillacea*	IMMI 1870	Eczema of integument	Germany
*R. argillacea*	IMMI 1881	Sputum from CF patient	Germany
*R. argillacea*	IMMI 1893	Sputum from CF patient	Germany
*R. argillacea*	IMMI 1894	Sputum from CF patient	Germany
*R. argillacea*	IMMI 1895	Sputum from CF patient	Germany
*R. piperina*	IMMI 1464	Sputum from CF patient	Germany

CBS, culture collection of the CBS-Fungal Biodiversity Centre, Utrecht, The Netherlands; DTO, internal culture collection of the CBS-Fungal Biodiversity Centre; IMMI, internal fungal collection of the Institute of Medical Microbiology, University Hospital Essen, Essen, Germany; CF, cystic fibrosis; BAL, broncho-alveolar lavage.

### Identification of clinical isolates

In addition to macro- and micromorphological features, the identification of clinical isolates was based primarily on sequence analysis of the internal transcribed spacer (ITS) 1 and two regions of the fungi, as was previously described for *Scedosporium* spp. 17.

### DNA extraction of cultured isolates

All fungal reference strains and clinical isolates were grown on malt extract agar (Oxoid, Wesel, Germany) at 35°C for 5 days. An agar block of approximately 1 cm^2^ in size was transferred to a 1.5-mL reaction tube and homogenized with a micropestle. The grounded mycelium–agar mixture was incubated at 56°C for 60 minutes with 500 μL of extraction buffer 18 containing 0.2 M Tris–HCl, 0.5 M sodium chloride, 0.01 M EDTA (pH 8.0), 1% SDS and 1 mg/mL proteinase K. After the addition of an equal volume of phenol (Roti-Phenol, Roth, Germany), the mixture was vigorously shaken and centrifuged at 20 000 *g* for 3 min. The supernatant containing the DNA was transferred to a new reaction tube and mixed with an equal volume of chloroform. After centrifugation at 20 000 *g* for 3 min, 0.2 volume of 5 M ammonium acetate and 0.7 volume of 2-propanol were added to the supernatant. The solution was incubated at room temperature for 10 min and then centrifuged at 20 000 *g* for 10 min. The resulting pellet of DNA was washed with 500 *μ*L of 70% ethanol, centrifuged at 20 000 *g* for 2 min, and air-dried. The dry pellet was re-suspended in 100 *μ*L of Tris-EDTA (TE) buffer (pH 8.0) and stored at 4°C until use. DNA concentration was measured with a Nanodrop 1000 spectrophotometer (Thermo Scientific, Dreieich, Germany).

### DNA extraction of respiratory specimens from CF patients

Samples were prepared with the MycXtra DNA preparation kit (Myconostica, Cambridge, UK) according to the manufacturer's instructions. To homogenize the respiratory samples, all specimens were pre-treated with an equal volume of freshly prepared Sputasol (Oxoid, UK) at 37°C for 15–30 min. The DNA from 1 mL of homogenized sputum was extracted. The elution volume was 80 μL in TE buffer; for PCR, tenfold serial dilution was again used.

### Primers and probe design

Primers and probe were designed by PrimerDesign™ Ltd. (Southampton, UK). Because of copyright issues, the sequences cannot be provided. All primers and probes bound within a 499-bp-long partial sequence of the calmodulin (CAL) gene of *Rasamsonia argillacea* (CBS 128787, GenBank accession number JF417504). The final amplification product was 87 bp in length.

### Real-time PCR assay

Amplification was performed on a Rotor-Gene 6000 thermal cycler in a final volume of 20 μL (Path-G. argillacea and Path-G. argillacea-standard, respectively (http://www.genesig.com/products/9254). The reaction mixture consisted of 10 μL of 2× Precision MasterMix (PrimerDesign™), 1 μL of *R. argillacea* pimer/probe mix, 1 μL of internal extraction control primer/probe mix, 3 μL of nuclease-free water, and 5 μL of sample or fungal DNA. The internal extraction control primer/probe mix was substituted by nuclease-free water whenever extracted DNA of culture strains was amplified. Specific amplification of the CAL gene was monitored in the FAM channel of the Rotor-Gene 6000 instrument, whereas inhibition control was monitored in the JOE channel.

### Assay detection limit

Serial ten-fold dilutions of the positive control (2 × 10^5^ to 2 × 10^0^ copies/μL of the CAL gene) were incorporated in the PCR kit. Ten-fold serial dilutions of DNA extracts from reference strains were made in TE buffer (pH 8.0). The detection limit was determined by comparing the final cycle threshold values of the sample dilutions with a standard curve established with dilutions of the positive control.

### Study cohort and mycological examination of respiratory samples from CF patients

In total, 214 samples from 138 CF patients (one to four samples per patient) attending the University Hospital Essen and the Clinic of the Ruhr, West German Lung Centre, Essen, Germany, in 2012 were tested for the presence of DNA from *Rasamsonia* species as mentioned above. For culturing, 100 μL of the sputasol-pretreated respiratory samples were inoculated on malt agar (Oxoid) and incubated aerobically at 36°C for 2 days and then at 22°C for an additional 8 days.

In addition, 20 sputum samples from 15 CF patients followed up in Angers University Hospital, Angers, France, were also analysed. Mycological examination of these samples performed in parallel to the PCR assay consisted of the inoculation of 10-μL aliquots of the digested samples on CHROMagar Candida (Becton-Dickinson, Franklin, NJ, USA), yeast extract-peptone-dextrose agar (YPDA) supplemented with chloramphenicol and gentamicin (Becton-Dickinson), Dichloran-rose bengale-chloramphenicol agar (DRBC), YPDA supplemented with chloramphenicol and cycloheximide, and erythritol agar. Incubation was carried out at 37°C for 2 weeks, except for the last two culture media, which were incubated at 25°C. Mould isolates were identified by cultural and microscopic morphological characteristics.

## Results

### Validation of the real-time PCR assay on cultured strains or isolates

The multiplex PCR assay did not demonstrate cross-reactivity with any of the 39 fungal or bacterial isolates shown in Table1.

**Figure 1:**
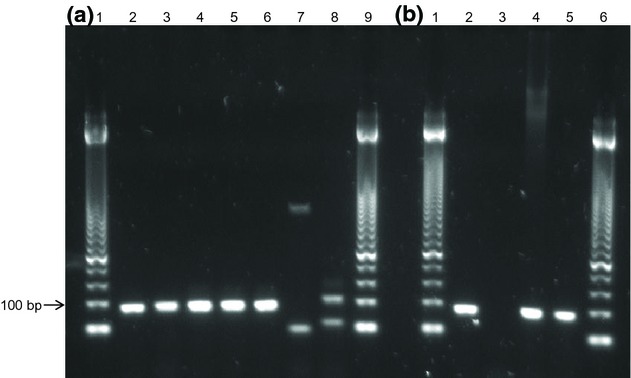
Results of the real-time PCR assay. (a) Results obtained with DNA extracts from pure cultures of reference strains: Lane 1, 50-bp DNA marker; Lane 2, *Rasamsonia argillacea* reference strain; Lane 3, *R. eburnea*; Lane 4, *R. aegroticola*; Lane 5, *R. piperina*; Lane 6, *R. argillacea*; Lane 7. *R. cyclindrospora*; Lane 8, *R. brevistipitata*; Lane 9, 50-bp DNA marker. (b) Results obtained with DNA extracts from sputum samples of positive samples from patients with cystic fibrosis (CF): Lane 1, 50-bp DNA marker; Lane 2, *R. argillacea* reference strain; Lane 3, sputum negative for *R. argillacea* complex; Lane 4, *R. argillacea* from CF patient 1; Lane 5, *R. aegroticola* from CF patient 2; Lane 6, 50-bp DNA marker.

The primer specificity of the assay was demonstrated by the fact that the amplification products from real-time PCR of six *Rasamsonia* species produced species-specific band sizes (87 bp) in a gel for all four species from the *R. argillacea* species complex (*R. argillacea*, *R. piperina*, *R. aegroticola* and *R. eburnea*) (Fig.1a). DNA from *R. brevistipitata* and *R*. *cylindrospora* could be amplified, but showed no specific band size of 87 bp.

**Figure 2:**
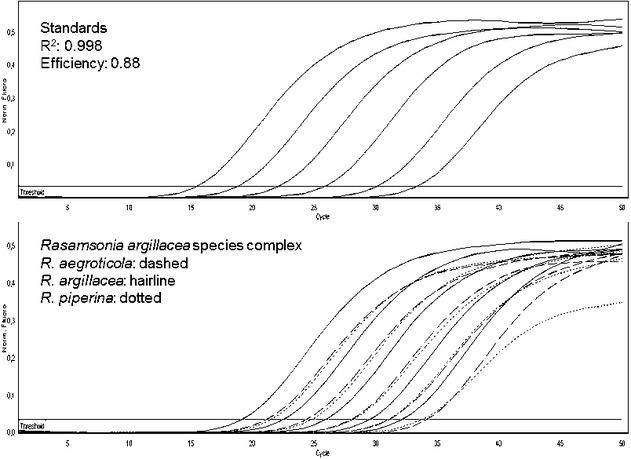
Amplification curves in the FAM channel for standards and DNA extracts from species of the *Rasamsonia argillacea* species complex demonstrating detection of *R. aegroticola*, *R. argillacea* and *R. piperina*. A tenfold serial dilution of the internal positive control (2 × 10^5^/μL to 2 × 10^0^/μL) was used as standard.

In contrast, DNA of *R. eburnea* was amplified by the primer set, but the probe of the assay was not able to detect this species in the FAM channel. The performance of the real-time PCR assay, using serial dilutions from the standard and from the extracted DNA of the three main species in the *R. argillacea* complex, is depicted in Fig.2. The detection limit was 7.51 copies/μL for *R.  aegroticola*, 2.51 copies/μL for *R. piperina* and 1.27 copies/μL for *R. argillacea* (∼0.01, 0.1 and 1 pg/μL of DNA). The assay was validated with a collection of 22 reference strains and 13 clinical isolates (Table2). All isolates of the three previously mentioned *Rasamsonia* species were detected, whereas reference strains of *R. eburnea*, *R. brevistipitata* and *R*. *cylindrospora* were not detected. In summary, the PCR assay is able to detect the three *Rasamsonia* species encountered in CF (*R. argillacea*, *R. piperina*, *R. aegroticola*) from the *R. argillacea* species complex.

### Validation of the real-time PCR assay with respiratory samples from CF patients

First, 214 respiratory secretions from 138 patients with CF were analysed to study the diagnostic utility of the *R. argillacea* species complex real-time PCR assay. No inhibition of PCR was observed in any sample.

For two patients, the results of the DNA assay were positive for *R. argillacea* complex DNA, and the assay generated an amplicon of the correct size (c.87 bp) in the gel (Fig.1b). Samples from one patient repeatedly tested were positive for *R. argillacea* by PCR, and *R. argillacea* was also detected by culture. The culture of the second PCR-positive sample was initially negative, but the patient became positive for *R. aegroticola* 5 months later (Table3).

**Table 3:** Characteristics of the cystic fibrosis (CF) populations tested by *Rasamsonia argillacea* species complex PCR and culture

CF population	University Hospital, Essen, Germany (*n* = 138)	University Hospital Angers, France (*n* = 15)
Mean age ± SD, years (range)	26.6 ± 10.1 (4–47)	17.1 ± 9.3 (3–33)
Female (%)	61 (44.2)	10 (66.7)
Number of specimens	214	20
Specimens per CF patient (range)	1.6 (1–4)	1.25 (1–4)
Specimens positive for the *R. argillacea* species complex		
By the real-time PCR assay	4	3
By cultures	3	2
By both PCR and cultures	3	2

To verify the results from this first single-centre approach, 20 sputa from 15 French CF patients were blindly analysed for the presence of *R. argillacea* complex DNA. Three samples from two patients were positive by PCR. In one patient, cultures from two successive sputum samples were positive for *R*. *argillacea* complex (Table3). The clinical and microbiological characteristics of all four CF patients with positive PCR for *R. argillacea* species complex are shown in Table4.

**Table 4:** Clinical and microbiological data of cystic fibrosis (CF) patients with DNA detection of *Rasamsonia argillacea* species complex

	Patient 1	Patient 2	Patient 3	Patient 4
Age, years	18	37	23	7
Sex	Male	Female	Female	Female
F508 del mutation	Homozygous	Homozygous	Homozygous	Heterozygous (W1204X)
Pancreatic insufficiency	No	No	Yes	Yes
CF-related diabetes	No	No	No	No
Listed for lung transplantation	No	No	No	No
FEV1	41%	30%	63%	80%
Clinical specimen tested	Sputum	Sputum	Sputum	Sputum
Cultures				
*Pseudomonas aeruginosa*	Yes	Yes	No	Yes
*Aspergillus fumigatus*	Yes	No	Yes	Yes
*R. argillacea* complex	*R. argillacea*	*R. aegroticola*	Yes	No

CF, cystic fibrosis; FEV1, forced expiratory volume in 1 seconds.

## Discussion

Here we describe the validation of a new, commercially available real-time PCR assay for the detection of three species from the *R. argillacea* complex. This assay was validated with both a collection of reference strains and clinical isolates of *Rasamsonia* species and was capable of detecting all three relevant species that are encountered in CF (*R*. *argillacea*, *R. piperina* and *R. aegroticola*). No other unrelated fungal or bacterial species were positive by the PCR assay. Of the clinical samples, two German CF patients were PCR-positive for the *Rasamsonia* species complex; one was initally positive for *R. argillacea* by culture whereas the culture from the other patient became positive for *R. aegroticola* 5 months later. Likewise, two of the 15 CF patients tested from France were found to be PCR-positive, but cultures detected the presence of the fungus for only one of them. These results may indicate that the PCR is more sensitive than culture.

*Rasamsonia* spp., formerly named *Geosmithia* spp., have been described as emerging pathogens in patients with CF 8,9. In total, four studies from France and the United Kingdom have reported the colonization of 18 CF patients by the *R. argillacea* species complex 8,9,19,20. However, the presence or persistence of these fungi was associated with a decrease in lung function or with pulmonary exacerbation in only one case 20. Our knowledge about the relevance and role of this fungus in CF is still limited because this pathogen has been only recently described, and data about its real prevalence in the CF population are lacking. The prevalence of the *R. argillacea* species complex in CF patients screened by the PCR assay in the present two-centre study was 2.6% (4/153). Recently, a single-centre study from Austria demonstrated that *R. argillacea* was found in five of 113 (4.4%) CF patients by culturing homogenized sputum samples 21.

In agreement with previous results, we found that colonization with *R. argillacea* complex was not associated with clinical deterioration in three out of the four PCR-positive patients. Conversely, a clinical and functional deterioration was seen for patient 3 (Table4), associated with the detection of the *R. argillacea* species complex by culture, but cultures also yielded numerous colonies of *Exophiala dermatitidis*.

When patients are immunocompromised (e.g. after lung transplantation), the *R. argillacea* complex can be relevant for CF patients, because it can cause invasive infections as demonstrated by cases of disseminated *R. argillacea* infections in patients with chronic granulomatous diseases or with haematological malignancies 22–24. Very recently, a case report from the USA described the infection of a pulmonary and aortic graft with *R. argillacea* in an immunocompetent patient 25.

All reports of infections with *R. argillacea* complex and a recent review highlight the importance and relevance of adequate identification of these species 25. Identification of *Rasamsonia* species is difficult because their phenotypic and morphological characteristics are very similar to those of other fungi, such as *Penicillium* and *Paecilomyces* species 10,16,22,24. The correct identification of the *Rasamsonia* species complex is not only important for differentiation from non-pathogenic fungi but is also crucial for therapeutic aspects, because the susceptibility profile of *Rasamsonia* species is different from that of many other moulds. Isolates from the *R. argillacea* complex show high MICs for the triazole drugs itraconazole, voriconazole and posaconazole, and low MICs for the echinocandins such as caspofungin and micafungin 8,16. Therefore, an accurate identification of this species complex is essential for minimizing the delay in diagnosis and in the initiation of appropriate antifungal therapy that results from difficulties in correct identification.

It has been reported that molecular-based techniques such as sequencing should be performed for species identification of the *R. argillacea* complex in conjunction with conventional mycological methods 16,26. However, sequencing is only available in specialized laboratories. PCR assays are established techniques in most laboratories and offer an excellent alternative tool for the detection of fungal DNA without culture methods. In addition to their higher sensitivity, PCR tests are more rapid than culture-based methods and allow the direct detection of fungi in patient specimens.

The primers and probes were labelled by PrimerDesign Ltd and we cannot provide the primer sequence because of copyright issues. However, one advantage of the real-time PCR assay described and validated by this study is that it is now commercially available (http://www.genesig.com/products/9254). Hence, all laboratories have access to this PCR assay. Future studies can use this standardized PCR assay to detect the *R. argillacea* species complex.

A limitation of the study is that the two study sites used different culture methods and conditions for fungal detection. In addition, the number of clinical specimens from the CF patient population that were found positive for species of the *R. argillacea* species complex was low, as this was only a double-centre study, and only four patients were colonized or infected with species of the *R. argillacea* complex.

In conclusion, the real-time PCR assay seems to be useful for routine detection of the three *Rasamsonia* species encountered in CF (*R. argillacea*, *R. piperina*, *R. aegroticola*). This novel assay may increase our understanding of the occurrence, aetiology and epidemiology of the *R*. *argillacea* species complex in CF.

## Conflict of Interest

None declared.
